# Comparison of Methods for Detection of *Blastocystis* Infection in Routinely Submitted Stool Samples, and also in IBS/IBD Patients in Ankara, Turkey

**DOI:** 10.1371/journal.pone.0015484

**Published:** 2010-11-18

**Authors:** Funda Dogruman-Al, Zahide Simsek, Kenneth Boorom, Eyup Ekici, Memduh Sahin, Candan Tuncer, Semra Kustimur, Akif Altinbas

**Affiliations:** 1 Department of Medical Microbiology, Gazi University School of Medicine, Besevler, Ankara, Turkey; 2 Yildirim Beyazit Education and Research Hospital, Department of Gastroenterology, Diskapi, Ankara, Turkey; 3 Blastocystis Research Foundation, Corvallis, Oregon, United States of America; 4 Section of Gastroenterology, Department of Internal Medicine, Gazi University School of Medicine, Besevler, Ankara, Turkey; Charité-University Medicine Berlin, Germany

## Abstract

**Background:**

This study compared diagnostic methods for identifying *Blastocystis* in stool samples, and evaluated the frequency of detection of *Blastocystis* in patients with irritable bowel syndrome (IBS) and inflammatory bowel disease (IBD).

**Results and Discussion:**

From a set of 105 stool specimens submitted for routine parasitological analysis, 30 were identified as positive for *Blastocystis* by the culture method. From that group of 30 positives, Lugol's stain, trichrome staining, and an immunofluorescence assay identified 11, 15, and 26 samples as positive respectively. Using culture as a standard, the sensitivity of Lugol's stain was 36.7%, trichrome staining was 50%, and the IFA stain was 86.7%. The specificity of Lugol's stain was 91%, trichrome staining was 100%, and the IFA stain was 97.3%. In the group of 27 IBS and IBD patients, using all methods combined, we detected *Blastocystis* in 67% (18/27) of the patients. *Blastocystis* was detected in 33% (2/6) of IBD patients and 76% (16/21) of IBS patients. For comparison, trichrome staining alone, the method most frequently used in many countries, would have only identified *Blastocystis* infection in 29% (6/21) of the IBS patients. No parasitic co-infections were identified in the IBS/IBD patients. Most *Blastocystis-*positive IBS/IBD patients were over 36 with an average length of illness of 4.9 years.

**Conclusions:**

Most IBS patients in this study were infected with *Blastocystis*. IFA staining may be a useful alternative to stool culture, especially if stool specimens have been chemically preserved.

## Introduction

*Blastocystis* is a single-celled parasite that infects the lower gastrointestinal tract of humans and animals. It is one of the few enteric parasites with a prevalence that often exceeds 5% in the general population of developed countries [1], [2], and exceeds 40% in individuals with chronic gastrointestinal illness [3], [4], [5]. Symptomatic infection with *Blastocystis* has been associated with abdominal pain (88%), diarrhea (23%), and constipation (32%) [6]. Additional symptoms reported include vomiting (13%), fatigue (11%), headaches, skin rashes, joint pain, and psychiatric illness [2], [7]. The majority of symptomatic *Blastocystis* cases occur in immunocompetent individuals [8] in the absence of any parasitic co-infection [9], [10]. Clinical diagnosis of *Blastocystis* infection is customarily performed with microscopical examination of stained, chemically preserved stool specimens, despite that method's lack of sensitivity [11].

Researchers have noted the need for reliable tests for *Blastocystis* to diagnose patients and to distinguish therapies which eradicate the organism from those that provide temporary symptomatic improvement [12]. Prior studies have compared the sensitivity of conventional staining techniques to commercially available assays in coprological detection of other unicellular enteric protists, such as *Giardia lamblia*, *Cryptosporidium parvum*, and *Entamoeba histolytica* [13], [14], [15], [16]. Such studies have often suggested that conventional staining techniques fail to identify many infections.

Antibodies Inc. (Davis, California, USA) and Savyon Diagnostics (Israel) have announced an immunofluorescence (IFA) assay and ELISA test respectively for the detection of *Blastocystis*. At the time of this study, only the IFA assay was available for evaluation. Following the methodology of prior studies of enteric protozoa, we compared the results obtained through the use of different diagnostic methods for *Blastocystis* when applied to stool samples submitted for routine parasitological analysis [13], [14], [15]. Prior studies have suggested that stool culture may be the most sensitive method available for *Blastocystis* [10], [17], so we used stool culture as a standard against which to evaluate the sensitivities of other detection methods.

### Investigation of *Blastocystis* in IBS and IBD patients

Irritable bowel syndrome (IBS) and inflammatory bowel disease (IBD) are chronic gastrointestinal illnesses of unknown causes which are common in developed countries. Symptoms of IBS are abdominal pain (100%), diarrhea (25%), and constipation (24%) [18]. Additional symptoms include vomiting, fatigue, headaches, and psychiatric symptoms [7]. IBS is the seventh most frequent diagnosis given to patients visiting primary care physicians [19], and studies of IBS prevalence have reported that 20–50% of referrals to gastroenterologists are due to IBS [18]. Although IBS is frequently identified as a psychosomatic disease, a number of studies have reported that many or most IBS patients can be shown to carry well established pathogenic gastrointestinal protozoa, or protozoa which can be shown to cause similar gastrointestinal illness in experimental animal infection [20], [21], [22], [23], [24]. *Blastocystis* has also been identified in association with intestinal inflammation [25] and IBD [26], [27], [28]. In the second part of the study, we tested stool samples from a group of IBS and IBD patients for *Blastocystis* using culture, conventional staining and IFA staining.

Neurological symptoms in IBS patients have traditionally been attributed to psychiatric causes, but a series of studies has identified an organic etiology by demonstrating that colonic biopsies from IBS and IBD patients secrete high levels of a trypsin-like serine protease [29]. Supernatants from the biopsies produced neurological symptoms when introduced into gastrointestinal tracts of mice by hypersensitizing neurons through the protease-activated (PAR2) pathway. The elevated trypsin-like protease production was found in all diarrhea-predominant and constipation-predominant IBS patients and IBD patients in multiple geographies by different research groups [29], [30], [31], but is absent in patients with viral and bacterial enteritis [31]. Researchers have not determined if the trypsin-like protease is endogenous or microbial in origin. In this study, we investigated biopsies from IBS and IBD patients for the presence of *Blastocystis* infection to try to evaluate the etiology of the trypsin-like protease.

## Materials and Methods

### Study subjects and specimens

For the evaluation of assay sensitivity and specificity, 105 stool samples submitted for parasitological analysis to the Gazi University School of Medicine were evaluated. As is consistent with prior studies in the evaluation of assay performance [13], [14], [15], the health histories from patients submitting those samples were not a focus of this study, but participant age, sex, year of onset, and illness has been included in supplemental file S1.

The second part of the study followed the methodologies used in prior studies which have evaluated the prevalence of *Blastocystis* infection [5], [22], [32] or other physiologically relevant phenomenon [29] in patients with illness diagnosed as IBS or IBD. Patients undergoing clinical evaluation at the Gazi University School of Medicine and Yildirim Beyazit Education and Research Hospital, Department of Gastroenterology, Ankara, Turkey were invited to participate in the study. IBS patients satisfied the Rome III criteria [33]. IBD patients were diagnosed with ulcerative colitis or Crohn's disease by clinical manifestation, sigmoidoscopy, barium enema, colonoscopy and laboratory analysis [34].

#### Colonoscopy Procedure

The colonoscopy procedure followed the method outlined in a prior study of trypsin production in IBS/IBD patients [29], except that midazolam (Dormicum: Roche) was used for sedation. Four biopsies were taken from the rectum. Two biopsies each were taken from the ascending, transverse, and descending colon.

#### Fecal culture

Unpreserved fecal specimens were cultured in Ringer's solution containing 10% horse serum and 0.05% asparagine at 37°C for 3–4 days, and screened with direct microscopy.

#### Conventional Staining

Fecal specimens were evaluated with Lugol's stain and trichrome staining [35].

#### IFA Staining

Fecal specimens were evaluated with Blasto-Fluor (Antibodies Inc., Davis, California), a commercially available immunofluorescence antibody (IFA) stain specific for *Blastocystis*, which was a gift from the manufacturer. Blasto-Fluor staining was performed by combining 200 µl of the fecal sample, 200 µl of phosphate buffered saline (PBS) and 4 µl of stain, then incubating for 60 minutes at 37°C. The sample was viewed under a fluorescence microscope using a 495 nm excitation filter and a 515 nm barrier filter. According to the manufacturer, the Blasto-Fluor stain was prepared from whole cell *Blastocystis* antigen from an American Tissue and Culture Collection (ATCC) culture identified as *Blastocystis* sp. subtype 3. The stain was prepared using a method similar to that reported in a 1993 United States National Institutes of Health paper [36]. The manufacturer's data sheet indicated no cross-reactivity to *E. histolytica*, *Giardia lamblia*, *Cryptosporidium parvum*, *Dientamoeba fragilis*, or *Saccharomyces cerevisiae*, and the NIH paper noted lack of cross-reactivity with “bacterial, fungal, and mammalian cells” [36]. We verified the stain showed no cross reactivity with *Giardia lamblia*, *Entamoeba coli*, *Candida*, *human leukocytes*, *and human erythrocytes*.

#### Biopsy Examination

Colonic biopsies were evaluated with the IFA stain as described previously in the procedure for stool samples. The biopsies were then compressed under a cover slip, and examined under a fluorescence microscope using the method described previously for stool specimens.

#### Statistical analysis

Statistical analysis was performed using the Statistical Package for the Social Sciences 13.0 software package. McNemar's test was applied to compare results from culture to other tests, and Pearson correlation coefficients were reported for all comparisons. Fisher's Exact Test was used to assess the significance of relationship between *Blastocystis* and IBS. A probability value of less than 0.05 was considered statistically significant.

#### Ethics and conflict of interest

Written informed consent was obtained from all study subjects. The study was approved by the Gazi University School of Medicine's Local Ethical Committee. All laboratory work was performed at the Department of Medical Microbiology Laboratory, Gazi University School of Medicine. Antibodies Inc. provided the IFA stain used in the study, but otherwise did not participate in or influence the design of the study, handling of samples, interpretation of the data, or the manuscript preparation.

## Results

In the collection of 105 stool samples, *Blastocystis* was detected in 28.6% (30/105) of the samples with stool culture (File S1). Of the 30 samples positive by culture, Lugol's stain, trichrome staining, and IFA staining identified 11, 15, and 26 samples as positive respectively. Using culture as a standard, the sensitivity of Lugol's stain was 36.7%, trichrome staining was 50%, and the IFA stain was 86.7%. The specificity of Lugol's stain was 91%, trichrome staining was 100%, and the IFA stain was 97.3%. Sensitivity was calculated as TRUE_POSITIVES/(TRUE_POSITIVES + FALSE_NEGATIVES). Specificity was calculated as TRUE_NEGATIVES/(TRUE_NEGATIVES + FALSE_POSITIVES). Thus, a test with no false negatives would have a sensitivity of 100%, while a test with no false positives would have a specificity of 100%. File S2 contains cross-tabulation tables with McNemar's test and Pearson correlation data.

The IBS/IBD patient group consisted of 27 patients, 21 with IBS and 6 with IBD, with 17 females, and 10 males. Patients ranged in age from 19 to 76 with a median age of 42. The earliest date of illness onset reported was 1995, and the most recent date was 2009. The average length of illness was 4.9 years. Most patients reported illness beginning after 2000. Using all methods combined, we detected *Blastocystis* in 67% (18/27) of the patients (Table 1). *Blastocystis* was detected in 33% (2/6) of the patients with IBD, and in 76% (16/21) of IBS patients.

**Table 1:** *Blastocystis* testing results from patients with IBS and IBD.

					*Blastocystis detected in stool with…*					
#	Code	Age	Sex	Disease	Year of onset	Lugol's stain, #cells/field	*Trichrome* staining	*Culture*	IFA stain, #cells/field	*BL* detected in colonic biopsy with IFA stain, #cells/field
1	Z-10	44	F	IBS-D	2009	4-5	+	+	+	-
2	Z-11	42	M	IBS-D	2008	-	-	-	0-1	+
3	Z-18	38	M	IBS-D	2008	-	-	+	-	-
4	Z-25	56	M	IBS-D	2006	-	-	-	S	-
5	G-3	26	M	IBS-D	2008	-	-	+	-	-
6	G-12	39	F	IBS-D	2009	-	-	+	10-15	0-1
7	Z-1	36	M	IBS-C	2000	3-4	+	+	-	-
8	Z-4	46	F	IBS-C	2004	-	-	-	-	-
9	Z-5	52	M	IBS-C	1998	S	-	-	-	-
10	Z-7	56	F	IBS-C	2001	-	-	-	-	-
11	Z-12	46	F	IBS-C	2002	1-2	+	-	4-6	-
12	Z-32	26	M	IBS-C	2006	-	-	-	-	-
13	Z-59	47	F	IBS-C	1995	-	-	-	-	S
14	G-15	40	F	IBS-C	2008	-	+	+	8-10	-
15	Z-3	50	F	IBS-A	2006	-	-	-	-	-
16	Z-9	29	F	IBS-A	2007	S	-	-	-	-
17	Z-16	25	F	IBS-A	2008	-	-	-	-	0-1
18	Z-21	44	F	IBS-A	2007	0-1	+	+	0-1	-
19	Z-49	40	F	IBS-A	2008	2-3	+	+	10-15	-
20	G-9	38	M	IBS-A	2006	-	-	-	-	-
21	G-11	48	F	IBS-A	2007	1-2	+	+	2-3	-
22	Z-36	46	M	IBD (UC)	2009	4-5	+	+	5-8	-
23	G-4	19	F	IBD (UC)	2008	-	-	-	-	-
24	G-21	76	M	IBD (UC)	1996	0-1	-	-	-	1
25	G-13	55	F	IBD	2000	-	-	-	-	-
26	G-14	27	F	IBD	2008	-	-	-	-	-
27	G-17	40	F	IBD	2006	-	-	-	-	-
Total						10	8	10	10	5

Numbers shown represent numbers of organisms per field. IBS-D, IBS-C and IBS-A refer to diarrhea-predominant IBS, constipation predominant IBS, or alternating diarrhea/constipation IBS. UC refers to Ulcerative Colitis. S = Organisms identified which are suspected to be *Blastocystis*, but were difficult to identify due to a distorted morphological form or light staining. These were counted as positives in compiling statistics.

While this study did not include a healthy control group, prior study performed at this site with Lugol's staining has identified the prevalence of *Blastocystis* infection in healthy controls at 11.6% (5/43) [1]. Comparing the results from Lugol's staining, we identified a statistically significant increase in *Blastocystis* infection in IBS patients over healthy controls, with 38% (8/21) of the IBS patients having *Blastocystis* infection vs. 11.6% (5/43) of the healthy controls (p = 0.0208, Fisher's Exact Test, two-tailed) [1].

Examination of biopsies generally failed to show *Blastocystis*, even in patients with positive stool tests. However one IBS patient (4.7%, 1/21) tested negative for *Blastocystis* by all coprological methods, while the biopsy showed the presence of *Blastocystis* (Table 1, patient Z-16).

No other parasitic infections, such as *Giardia lamblia* or *E. histolytica*, were detected in trichrome staining of stool samples from the IBS/IBD group.

## Discussion

We were able to identify *Blastocystis* in patients using common laboratory methods. But a standard method used in clinical practice, trichrome staining, did not perform well. The low sensitivity of this method has been reported in prior studies from Thailand [17], United Kingdom [37], Denmark [10], and the United States [38]. Prior studies have attributed the difficulty of identifying *Blastocystis* to its large number of morphological forms [11], the large size variation exhibited by the organism [7], or its similarity in appearance to a fat cell [11].

### Comparison of techniques

In our study, relative to stool culture, the IFA stain provided a sensitivity of 86.7% and specificity of 97.3%, an improvement over trichrome staining's sensitivity of 50% and specificity of 100%. The IFA stain has the advantage of providing a result in approximately one hour, instead of the 3–4 days required for culture. The method does not require the training associated with stool culture, trichrome staining or PCR analysis.

**Figure 1:**
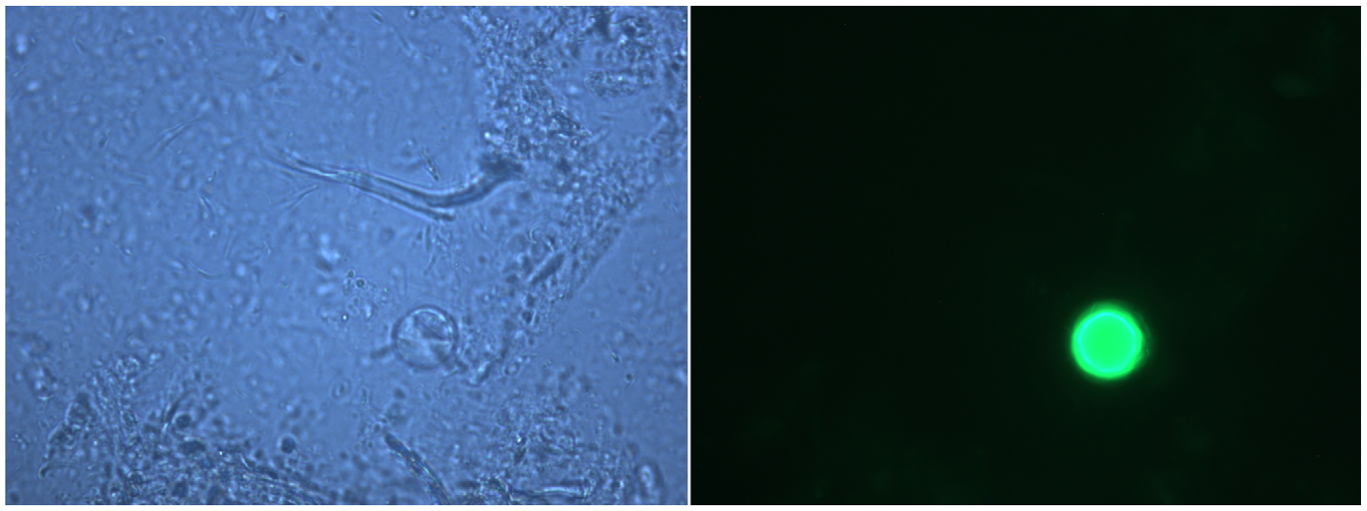
Stool sample stained with IFA stain, comparing bright field (left) and fluorescence microscope view (right) showing *Blastocystis*. High resolution photomicrographs courtesy of Antibodies Inc.

Molecular diagnostics like this one, which provide a clearer visual indicator of positive or negative status (figure 1), may help provide some consistency between different labs, possibly addressing regional disagreements [7] concerning the importance of the organism in disease. A comparison among European reference laboratories found that *Blastocystis* was the most inconsistently detected enteric protozoan. From a shared set of 102 samples, one reference laboratory identified 25 samples as *Blastocystis-*positive, while another reference laboratory identified 90 samples as *Blastocystis-*positive [39]. A similar study between two European diagnostic centers with sub-specialties in tropical medicine reported that when each was given a set of 48 stool samples prepared to be identical in parasitological content, one center identified 29 as *Blastocystis*-positive, while the other identified 38 as positive. A further comparison between one of the European diagnostic centers and a West African center using 78 stool samples collected from identical patients but on different days found that the European center identified 38 as *Blastocystis*-positive, while the West African center identified 7 as positive [40]. Substantial differences were also found in the detection of *Entamoeba histolytica/dispar*, though not as great as those seen in the detection of *Blastocystis*.

Staining of formalin-preserved specimens (vs. unpreserved specimens) with the IFA stain appeared to produce no loss in sensitivity (data not shown). The culture and PCR methods have not been shown to be compatible with chemically preserved specimens. While we did not test the stability of the stool antigen, the IFA stain's manufacturer reported that 6-month old formalin-preserved stool specimens stain successfully. The IFA assay offers cost advantages over PCR [10], although it does not provide information about *Blastocystis* genotype. While the IFA method offers an improvement over existing laboratory techniques, it was still unable to detect some infections, possibly because it requires the presence of whole or partial cells in the sample.

Some culture-positive samples were judged negative on the IFA staining portion of the study, however closer examination showed the presence of lightly stained, irregularly formed cells. A detailed investigation of the phenomenon is beyond the scope of this study.

No single diagnostic method could have identified all of the infections found in IBS patients (Table 1), although stool culture would have identified the largest number of infections we found. PCR has been suggested for clinical diagnosis of *Blastocystis* infection [10], but PCR testing offers challenges as well, as a prior *Blastocystis* study found that PCR failed to detect 25% of samples positive by culture due to PCR inhibitors in stool samples [41]. A lower sensitivity of PCR compared to stool culture was also found in a Pakistani study of IBS patients [22]. Some inherent limitations may exist in the use of coprological tools to investigate *Blastocystis* infection: experimental infection of mice has suggested *Blastocystis* cells may embed themselves deep in the gastrointestinal tract, reaching muscle layers [42], and also survive for long periods of time in immunocompetent animals outside of the gastrointestinal tract [43].

### *Blastocystis* in IBS patients

We report that most IBS patients (76%) in our study were infected with *Blastocystis*, which we previously showed generally consists of subtypes 2 and 3 in Ankara, Turkey [26]. The finding of elevated frequency of *Blastocystis* infection is similar to prior studies from Pakistan [3], the United Kingdom [4], and Italy [32] (Table 2). A recent study from Pakistan identified *Blastocystis* infection in 53% (90/171), and *Dientamoeba fragilis* in 4% (7/171) of diarrhea-predominant IBS patients by stool culture [22]. That study specifically excluded patients with *Giardia lamblia*, *Entamoeba histolytica*, *Salmonella* spp., *Campylobacter jejuni*, *Clostridium difficile*, and *Vibrio cholerae*. Our finding of symptomatic *Blastocystis* mono-infection is consistent with prior research from Canada [8], Denmark [10], France [41], Egypt [41], Australia [44], Jordan [45], and the United States [38], [46].

**Table 2:** Results from studies of *Blastocystis* infection in IBS patients in Europe and the Middle East.

Country	Year	*Blastocystis* detection method	Frequency of detection in IBS patients	Frequency of detection in controls
Turkey (this study)	2010	Lugol stain	38% (8/21)	11.6% (5/43)
Pakistan [5]	2010	Stool culture	60% (95/158)	24% (38/157)
United Kingdom [63]	2005	Trichrome stain	38% (>800 samples)	7% [64]
Pakistan [3]	2004	Stool culture	46% (44/95)	7% (4/55)
Italy [32]	1999	Formalin-ether concentration	18.5% (15/81)	7.5% (23/307)

The UK study included patients with other types of chronic gastrointestinal illness.

Prior study has suggested that the type of disease found in IBS and IBD patients is dissimilar to bacterial or viral enteritis, as patients with the later diseases do not express the high levels of trypsin-like compound that are found in fecal specimens from IBS/IBD patients [31]. As the production of such a trypsin-like compound by colonic biopsies is present in all IBS/IBD patients, but is of unknown origin [29], [30], [31], the biopsy data from this study may help elucidate the source of that compound. Since most biopsies did not show *Blastocystis*, we suggest that this organism is an unlikely source for the trypsin. A number of tissues in the human body have been recently found to produce trypsin-like compounds. The production may be related to the innate immune activation found in IBS/IBD patients [47], [48], [49].

### *Blastocystis* in IBD patients

We detected *Blastocystis* infection in a third of the IBD patients, although the size of the IBD group was small. A prior study from Mexico identified *Blastocystis* in 10% of IBD patients [27], and noted presence of *Blastocystis*, *E. histolytica*, or *Entamoeba coli* was correlated with relapsing illness [27].

### Parasite quantity in *Blastocystis* infection

*Blastocystis* is the only common intestinal parasite where the number of organisms per field is represented as medically significant [7], and studies have suggested that *Blastocystis* simply “overgrows” in response to some other type of illness. We were unable to demonstrate any kind of consistent *Blastocystis* overgrowth in these IBS/IBD patients. Although *Blastocystis* was found consistently, few of the IBS and IBD patients had large quantities of *Blastocystis* in stool samples. Parasite quantity in stool samples is generally uncorrelated with symptomatic severity in enteric infections in humans, so sensitive PCR and ELISA tests are employed to diagnose diseases such as giardiasis or ameobiasis [50]. Other papers have addressed the mechanisms underlying such symptomatic expression in protozoal illness [51], [52], [53]. A review of research is beyond the scope of this paper, however pathogenic protozoa express ligands which become physiologically reactive at low concentrations [51], [52], [53] in hosts that do not express IgA antibodies to them. Hosts may fail to express IgA due to lack of prior exposure, or the presence of host genetic mutations in Toll-like receptor genes and cytokine expression, such as those which have been found in IBS and IBD patients [54], [55]. Protozoal ligands may interfere with the host's adaptive immune response, producing long-term infection and symptoms [52], [56].

### Clinical Significance of *Blastocystis* in IBS Patients

*Blastocystis* was associated with IBS as early as 1986, when a US study attributed gastrointestinal illness in patients mono-infected with *Blastocystis* to IBS, in support of the hypothesis that *Blastocystis* was non-pathogenic [57]. By 1997, researchers had begun reporting the appearance of *Blastocystis* infection at statistically significant rates in patients with IBS and chronic gastrointestinal illness in Pakistan [3], Italy [32], and the United Kingdom [4], with public health officials urging that *Blastocystis* be excluded before diagnosing patients with IBS [58]. IBS “outbreaks” have included returning international travelers to the US [59], a community exposure to contaminated water in Canada [60], [61], and IBS-like illness in association with military service in the Persian Gulf [62].

Host factors, such as age, have been suggested as contributing to the expression of symptoms in *Blastocystis* infection [7], [26]. In our study, 68% (19/28) of the IBS/IBD patients were between the ages of 38 and 56. The median age of the study's patient population was 42, 15 years older than the median age of Turkey's population. While the age distribution of participants in a voluntary study can be influenced by self-selection, a number of other studies have noted age relationships. A study of *Blastocystis* in 158 diarrhea-predominant (IBS-D) patients in Pakistan [5] reported a mean age of 41. A histogram describing symptomatic *Blastocystis* infection in a 1990 Canadian study of 143 patients showed a peak in infection rates in patients who were 33–34 years of age [8]. Prior studies have noted differences in symptomatic expression of *Blastocystis* based on age [45].

### Conclusions

Most *Blastocystis* infections are not detectable in stool samples with conventional staining techniques, and most IBS patients in this study were infected with *Blastocystis*. Stool culture and IFA staining provide better sensitivity than conventional staining techniques, but still fail to detect infection in some symptomatic patients. IFA staining provides a more sensitive method than conventional staining for identifying *Blastocystis* infection in chemically preserved stool samples.

## Supporting Information

File S1 Testing results and patient data on study subjects. (XLS)

File S2 Crosstabs of results from different methods of Blastocystis testing. (DOC)
