# Supplementary material for: Comparison of Methods for Detection of Blastocystis Infection in Routinely Submitted Stool Samples, and also in IBS/IBD Patients in Ankara, Turkey
Source: PLoS One. 2010 Nov 18;5(11):e15484. doi: 10.1371/journal.pone.0015484 (PMC2987810; doi:10.1371/journal.pone.0015484)
Supplement: File S2 — Crosstabs of results from different methods of Blastocystis testing. (DOC) [file pone.0015484.s002.doc]

| **NativeLugol (NL) * Culture Crosstabulation** | | | | | |
| --- | --- | --- | --- | --- | --- |
|  |  |  | Culture | | |
|  |  |  | negative | positive | Total |
| NL | negative | Count | 70 | 19 | 89 |
|  |  | % within NL | 78,7% | 21,3% | 100,0% |
|  | positive | Count | 5 | 11 | 16 |
|  |  | % within NL | 31,2% | 68,8% | 100,0% |
|  | Total | Count | 75 | 30 | 105 |
|  |  | % within NL | 71,4% | 28,6% | 100,0% |

| **Test Statistics^b^** | |
| --- | --- |
|  | NL & Culture |
| N | 105 |
| Exact Sig. (2-tailed) | ,007^a^ |
| a. Binomial distribution used. | |
| b. McNemar Test | |

| **Trichrom * Culture Crosstabulation** | | | | | |
| --- | --- | --- | --- | --- | --- |
|  |  |  | Culture | | |
|  |  |  | negative | positive | Total |
| Trichrom | negative | Count | 75 | 15 | 90 |
|  |  | % within Trichrom | 83,3% | 16,7% | 100,0% |
|  | positive | Count | 0 | 15 | 15 |
|  |  | % within Trichrom | ,0% | 100,0% | 100,0% |
|  | Total | Count | 75 | 30 | 105 |
|  |  | % within Trichrom | 71,4% | 28,6% | 100,0% |

| **Test Statistics^b^** | |
| --- | --- |
|  | Trichrom & Culture |
| N | 105 |
| Exact Sig. (2-tailed) | ,000^a^ |
| a. Binomial distribution used. | |
| b. McNemar Test | |

| **FITC * Culture Crosstabulation** | | | | | |
| --- | --- | --- | --- | --- | --- |
|  |  |  | Culture | | |
|  |  |  | negative | positive | Total |
| FITC | negative | Count | 73 | 4 | 77 |
|  |  | % within FITC | 94,8% | 5,2% | 100,0% |
|  | positive | Count | 2 | 26 | 28 |
|  |  | % within FITC | 7,1% | 92,9% | 100,0% |
|  | Total | Count | 75 | 30 | 105 |
|  |  | % within FITC | 71,4% | 28,6% | 100,0% |

| **Test Statistics^b^** | |
| --- | --- |
|  | FITC & Culture |
| N | 105 |
| Exact Sig. (2-tailed) | ,687^a^ |
| a. Binomial distribution used. | |
| b. McNemar Test | |

| **FITC * Trichrom Crosstabulation** | | | | | |
| --- | --- | --- | --- | --- | --- |
|  |  |  | Trichrom | | |
|  |  |  | negative | positive | Total |
| FITC | negative | Count | 76 | 1 | 77 |
|  |  | % within FITC | 98,7% | 1,3% | 100,0% |
|  | positive | Count | 14 | 14 | 28 |
|  |  | % within FITC | 50,0% | 50,0% | 100,0% |
|  | Total | Count | 90 | 15 | 105 |
|  |  | % within FITC | 85,7% | 14,3% | 100,0% |

| **Test Statistics^b^** | |
| --- | --- |
|  | FITC & Trichrom |
| N | 105 |
| Exact Sig. (2-tailed) | ,001^a^ |
| a. Binomial distribution used. | |
| b. McNemar Test | |

| **Native Lugol (NL) * FITC Crosstabulation** | | | | | |
| --- | --- | --- | --- | --- | --- |
|  |  |  | FITC | | |
|  |  |  | negative | positive | Total |
| NL | negative | Count | 72 | 17 | 89 |
|  |  | % within NL | 80,9% | 19,1% | 100,0% |
|  | positive | Count | 5 | 11 | 16 |
|  |  | % within NL | 31,2% | 68,8% | 100,0% |
|  | Total | Count | 77 | 28 | 105 |
|  |  | % within NL | 73,3% | 26,7% | 100,0% |

| **Test Statistics^b^** | |
| --- | --- |
|  | NL & FITC |
| N | 105 |
| Exact Sig. (2-tailed) | ,017^a^ |
| a. Binomial distribution used. | |
| b. McNemar Test | |

| **sex * Culture Crosstabulation** | | | | | |
| --- | --- | --- | --- | --- | --- |
|  |  |  | Culture | | |
|  |  |  | negative | positive | Total |
| sex | male | Count | 30 | 17 | 47 |
|  |  | % within sex | 63,8% | 36,2% | 100,0% |
|  | female | Count | 45 | 13 | 58 |
|  |  | % within sex | 77,6% | 22,4% | 100,0% |
|  | Total | Count | 75 | 30 | 105 |
|  |  | % within sex | 71,4% | 28,6% | 100,0% |

| **Chi-Square Tests** | | | | | |
| --- | --- | --- | --- | --- | --- |
|  | Value | df | Asymp. Sig. (2-sided) | Exact Sig. (2-sided) | Exact Sig. (1-sided) |
| Pearson Chi-Square | 2,407^a^ | 1 | ,121 |  |  |
| Continuity Correction^b^ | 1,780 | 1 | ,182 |  |  |
| Likelihood Ratio | 2,401 | 1 | ,121 |  |  |
| Fisher's Exact Test |  |  |  | ,134 | ,091 |
| Linear-by-Linear Association | 2,384 | 1 | ,123 |  |  |
| N of Valid Cases | 105 |  |  |  |  |
| a. 0 cells (,0%) have expected count less than 5. The minimum expected count is 13,43. | | | | | |
| b. Computed only for a 2x2 table | |  |  |  |  |

| **Correlations** | | | |
| --- | --- | --- | --- |
|  |  | Culture | FITC |
| Culture | Pearson Correlation | 1,000 | ,858^**^ |
|  | Sig. (2-tailed) |  | ,000 |
|  | N | 105,000 | 105 |
| FITC | Pearson Correlation | ,858^**^ | 1,000 |
|  | Sig. (2-tailed) | ,000 |  |
|  | N | 105 | 105,000 |
| **. Correlation is significant at the 0.01 level (2-tailed). | | | |

| **Correlations** | | | |
| --- | --- | --- | --- |
|  |  | Culture | Trichrom |
| Culture | Pearson Correlation | 1,000 | ,645^**^ |
|  | Sig. (2-tailed) |  | ,000 |
|  | N | 105,000 | 105 |
| Trichrom | Pearson Correlation | ,645^**^ | 1,000 |
|  | Sig. (2-tailed) | ,000 |  |
|  | N | 105 | 105,000 |
| **. Correlation is significant at the 0.01 level (2-tailed). | | | |

| **Correlations** | | | |
| --- | --- | --- | --- |
|  |  | Culture | NL |
| Culture | Pearson Correlation | 1,000 | ,377^**^ |
|  | Sig. (2-tailed) |  | ,000 |
|  | N | 105,000 | 105 |
| NL | Pearson Correlation | ,377^**^ | 1,000 |
|  | Sig. (2-tailed) | ,000 |  |
|  | N | 105 | 105,000 |
| **. Correlation is significant at the 0.01 level (2-tailed). | | | |
